# Colorectal Cancer Screening Knowledge and Practices Among Practicing Obstetrician-Gynecologists and Residents

**DOI:** 10.1089/whr.2022.0065

**Published:** 2023-01-05

**Authors:** Camille A. Clare, Clive Liu, Arielle Greenberg, Penny Liberatos, Lindsey Channen, Kavitha Ram, Silvia Fernandez, Jennifer Harley

**Affiliations:** ^1^ Department of Obstetrics and Gynecology, New York Medical College, Valhalla, New York, USA.; ^2^ New York Medical College, School of Medicine, Valhalla, New York, USA.; ^3^ Department of Public Health, New York Medical College, Valhalla, New York, USA.; ^4^ Department of Medicine, Icahn School of Medicine at Mount Sinai, New York, New York, USA.; ^5^ Department of Obstetrics and Gynecology, Jamaica Hospital Medical Center, Jamaica, New York, USA.; ^6^ Department of Gastroenterology, New York City Health + Hospitals/Metropolitan, New York, New York, USA.

**Keywords:** colorectal cancer screening, guidelines, obstetricians-gynecologists, adherence, knowledge

## Abstract

**Background:**

Colorectal cancer (CRC) is the second leading cause of cancer death in the US, the third most diagnosed cancer in women, and the second leading cause of cancer death in women. The aims of our study are to (1) investigate knowledge of and adherence to CRC screening guidelines by obstetrician-gynecologists (Ob/Gyns) and (2) assess whether this knowledge/adherence vary by demographic and practice characteristics.

**Methods:**

An anonymous cross-sectional survey was distributed to a convenience sample of 142 practicing Obs/Gyns drawn from National Medical Association section members/conference attendees and hospital Ob/Gyn department members.

**Results:**

Most respondents (80.3%) viewed colorectal screening within the scope of Ob/Gyn practice, and 71.8% used the American College of Obstetricians and Gynecologists guidelines for screening. Most respondents were knowledgeable regarding CRC screening but not in all areas. On average they only identified half of the 10 risk factors listed and only one-quarter correctly identified the age when screening can stop. Residents were somewhat more knowledgeable about screening guidelines and risk factors than attendings. More than half of respondents (57.8%) reported always initiating CRC screening for the appropriate age and risk factors. Respondents identified education and awareness (56.3%) and patients' unwillingness to undergo an invasive procedure (75.4%) as barriers to screening.

**Conclusions:**

Knowledge regarding CRC screening was less than optimal and differed by attending/resident status. Greater emphasis should be placed on CRC screening and guidelines training for primary care providers like Ob/Gyns. Some of this could be accomplished through maintenance of certification and continued integration into residency education.

## Introduction

Colorectal cancer (CRC) is the second leading cause of cancer death in the United States, the third most diagnosed cancer in women, and second leading cause of cancer death in women.^1^ CRC screening, including removing precancerous polyps at the time of screening, has helped to decrease the incidence of CRC and later stage disease. The top tier CRC screening options based on current recommendations from the US Multi-Society Task Force on CRC are (1) colonoscopy every 10 years and annual fecal immunochemical test (FIT); (2) computed tomography (CT) colonography every 5 years, FIT-fecal DNA test every 3 years; and flexible sigmoidoscopy every 5–10 years.^2^ The Task Force also recommended in 2017 that screening begin for most average-risk individuals at age 50 and at age 45 years for African-Americans.^2^ This recommendation was revised to age 45 years for everyone in 2022.^3^

According to the American Cancer Society, patients diagnosed with localized colon cancer have a 5-year survival rate of as high as 90% compared to those with later-stage regional (72%) or later-stage distant (14%) colon cancer.^4^ Therefore, appropriate CRC screening is crucial in reducing the morbidity and mortality rates of the disease through early detection. In 2020, among adults 50–75 years old, 71.6% had up-to-date CRC screening (based on USPTF guidelines) and 19.9% were never screened.^4,5^ Data from 2018 showed that a higher proportion of those 65–75 years old were reported to be up to date with CRC screening compared to those aged 50–64 (79.2% vs. 63.3%.^6^ Although these rates have been increasing over the last few years, they are still short of the Healthy People 2030 target of 74.4%.^7^

An important factor that contributes to the rate of CRC screening is the recommendation of the physician.^8,9^ In fact, ninety percent of patients who undergo colonoscopy do so at the recommendation of their physician.^10^ Over the last decade, the role of the primary care physician in CRC screening has been recognized to be increasingly important, especially since they tend to have more frequent contact with patients.^11^

Young women in their reproductive years often view their gynecologist as their most essential primary care physician for early preventative care, and for many, this continues well into late adulthood.^12,13^ In addition, there are certain high-risk patients who warrant earlier screening, such as those with first degree family members with CRC, and diagnoses, such as Lynch Syndrome, and Family Colon Cancer Syndrome X.^2^ This validates the significant role that gynecologists can play in recommending CRC screening, and the importance that they be aware of current guidelines.

Although obstetrician-gynecologists (Ob/Gyns) play an important role in cancer prevention, some may not be aware of and/or adhere to the current CRC screening guidelines. For example, one study in 2009 indicated that 55.4% of providers correctly identified the appropriate age at which to begin routine CRC screening for average risk patients.^13^ Another study showed that only 87.2% of Ob/Gyns ordered or performed CRC screening^10^ and it was ordered less often than both breast cancer and cervical cancer screenings. In addition, while 88%–95% of women were compliant with their breast and cervical cancer screening, only ∼66% of women in the appropriate age range (50+ years) were compliant with CRC screening.^14^

Despite this important role of Ob/Gyns, there have been very few studies that have focused on this group of primary care physicians and none within the last 10 years. Understanding where they may have knowledge gaps in identifying women who should be screened, using the most recent screening guidelines, and assisting patients to overcome barriers to screening can help identify the education needs of Ob/Gyns and patients in this area with the goal to reduce CRC.

The aims of our study are (1) to investigate knowledge of and adherence to CRC screening guidelines by Ob/Gyns and (2) to assess whether this knowledge and adherence varies by their demographic and practice characteristics.

## Materials and Methods

### Study design and participants

This study consisted of a cross-sectional survey of practicing Ob/Gyns and residents in Ob/Gyn training programs in the United States. The participants constituted a convenience sample drawn from four sources: (1) members of the National Medical Association (NMA) Obstetrics and Gynecology section; (2) attendees at the 2018 NMA national conference in Orlando, Florida; (3) attendees at the 2018 American College of Obstetricians and Gynecologists (ACOG) District II conference in New York City (NYC); and (4) members of the Department of Ob/Gyn at the NYC Health + Hospitals (H+H)/Metropolitan.

The NMA Ob/Gyn section members were sent an electronic survey based on email addresses of ∼300 section members. Paper surveys were distributed to attendees at the NMA national convention, ACOG District II conference, and to members of the Department of Ob/Gyn at the NYC H + H/Metropolitan. Although there were ∼150 attendees at the NMA conference, 100 at the ACOG district conference and about 50 hospital Ob/Gyn department members, the number of individuals who were approached or knew about the survey is not known. Institutional Review Board approval was obtained from the Biomedical Research Alliance of New York (BRANY) [17-12-446-182(HHC)].

### Measures

An anonymous questionnaire was developed by the authors, consisting of 17 closed-ended items (Supplementary Appendix A1). The topics covered by the questionnaire were developed based on a review of the literature. In addition, some questions were based on the recommendations of the American College of Gastroenterology and the American Gastroenterological Association.

Questionnaire topics included knowledge regarding CRC (*i.e.,* risk factors, recommended ages, screening intervals, reasons to screen earlier, and subsequent diagnostic steps after positive results); knowledge of components of recommended guidelines; screening methods used; screening guidelines used in practice; frequency of screening conversations with patients; patient barriers to screening; suggestions for patient adherence to screening; and demographic/practice characteristics. It should be noted that at the time of the survey, the guidelines recommended that CRC screening begins at age 50 years, which was the expected age used for the survey.^2^ It was also assumed that participants should know the risk factors and screening guidelines for CRC as would be expected for any primary care provider.^11^

CRC knowledge was specifically assessed in three areas, based on the identification of (1) risk factors for CRC from a list of 10 factors; (2) appropriate screening intervals for each of six screening methods; and (3) circumstances where they would recommend earlier screening from a list of six situations presented. The number of items correct in each of these three categories was calculated for each respondent.

Demographic and practice characteristics included gender identity (male/female) (nonbinary categories were not included); age group (20–50/>50); race/ethnicity (white/African American/Hispanic/Asian); academic rank (attending/fellow/resident/other); geographic location (Northeast/South/Midwest-West [NE/S/MW-W]); current practice type (hospital-based/academic-based/private practice/community-based); and current practice (obstetrics only/gynecology only/both obstetrics + gynecology). The questionnaire was reviewed for content validity by the authors (three gastroenterologists, three Ob/Gyns, one internal medicine resident) and pretested with six Ob/Gyn attendings for content and clarity of wording.

### Data collection

Data were collected between October 2018 and January 2019. Attendees at the NMA National Convention and at the ACOG District 2 conference were contacted in person at random and asked to complete paper surveys in between conference sessions by the primary author. Members of the Department of Ob/Gyn of NYC H+H/Metropolitan were asked in person to complete paper surveys during departmental meetings. In addition, electronic surveys were sent to the Ob/Gyn section members of the NMA during this time.

### Data analysis

Descriptive analyses consisted of frequencies, percentages, means, and standard deviations (as appropriate). Subset analyses with demographic and practice characteristics were conducted using chi-square analysis or Fisher's exact test (in the case of small cell sizes), *t*-tests, and one-way analysis of variance (ANOVA). Analyses were conducted in SPSS (v. 23) and Stata (v.15). *p*-values <0.05 were considered statistically significant.

## Results

A total of 142 respondents answered the survey. A response rate cannot be calculated since the number of individuals who knew about or were handed a paper survey at the conferences and department meetings is not known. Two-thirds of survey respondents were female and were evenly divided between those older and younger than the age of 50 years (Table 1). Over 60% were African American and one-quarter were white. Three-quarters were attendings or fellows and most (59.2%) were from the Northeast. About one-third of them were hospital-based and about one-quarter were academic-based, and a similar proportion were in private practice with two-thirds practicing both obstetrics and gynecology.

**Table 1.** Demographic Characteristics of Study Sample

Characteristic	*n*	%
Gender		
Male	42	32.3
Female	88	67.7
Age		
20–30	19	14.5
31–40	17	12.9
41–50	26	19.9
51–60	32	24.4
61+	37	28.3
Race/Ethnicity		
White	32	25.2
African-American	79	62.2
Hispanic	4	3.1
Asian	12	9.5
Academic rank		
Attending/fellow	93	75.0
Resident	20	16.1
Other	11	8.9
Geographic location		
Northeast	71	59.2
South	28	23.3
Midwest	17	14.2
West	4	3.3
Current practice type		
Hospital-based	38	34.6
Academic	28	25.5
Private practice	25	22.7
Community-based	19	17.3
Current practice		
Obstetrics only	16	13.3
Gynecology only	24	20.0
Both obstetrics + gynecology	80	66.7

### Knowledge regarding CRC screening

Of the 10 risk factors specified, the mean number correct was 5.3 (± 1.8) with only one in four participants correctly identifying at least seven risk factors (Table 2). Those risk factors most likely to be correctly identified (at least 75%) were familial adenomatous polyposis, first degree relative with CRC before age 60, HNPCC-Lynch syndrome, and inflammatory bowel disease. Inflammatory bowel syndrome, celiac disease, diabetes, and vegetarian diet were least likely to be identified correctly (<30%).

**Table 2.** Correct Responses Regarding Participant Knowledge of Colorectal Cancer Screening

Characteristic	*n*	%	Mean ± SD
Risk factors (correct)			
Familial adenomatous polyposis	137	96.5	
First-degree relative with CRC before age 60	126	88.7	
HNPCC-Lynch syndrome	124	87.3	
Inflammatory bowel disease	107	75.4	
Juvenile polyposis	88	62.0	
Peutz-Jeghers	60	42.3	
Inflammatory bowel syndrome	42	29.6	
Celiac disease	35	24.6	
Diabetes mellitus	26	18.3	
Vegetarian diet	1	0.7	
Risk factors (no. of items correct)			5.3 ± 1.8
0–3	24	16.9	
4–6	83	58.5	
7–10	35	24.6	
Recommended ages (correct)			
Begin screening			
Age 45 or 50	132	93.6	
End screening			
Age 75	33	23.2	
Both recommended ages	22	15.5	
Screening interval (correct)			
Fecal occult blood test (1 year)	126	88.7	
Colonoscopy (10 years)	109	76.8	
Flexible sigmoidoscopy (5 years)	95	66.9	
Fecal immunohistochemical test (1 year)	80	56.3	
Double contrast barium enema (5 years)	61	43.0	
CT colonography (5 years)	53	37.3	
Screening interval (no. of items correct)			3.7 ± 1.8
0–2	38	26.8	
3–4	51	35.9	
5–6	53	37.3	
Earlier screening (correct)			
Positive fecal occult blood test	120	93.0	
Family member with new CRC diagnosis	99	76.7	
New diagnosis of inflammatory bowel disease	86	66.7	
Change in stool pattern/frequency/appearance	83	64.3	
New diagnosis of other cancer	57	44.2	
New onset abdominal symptoms	54	41.9	
Earlier screening (no. of items correct)			3.9 ± 1.5
0–2	25	19.4	
3–4	60	46.5	
5–6	44	34.1	
Next diagnostic step after positive			
Result (correct)			
Colonoscopy	125	88.0	

CRC, colorectal cancer; CT, computed tomography; SD, standard deviation.

Almost all respondents knew the correct ages to initiate CRC screening, but fewer than one-quarter knew the correct age to end screening. Most were knowledgeable about the correct screening intervals for fecal occult blood test (FOBT) and colonoscopy but were less knowledgeable about those for double contrast barium enema and CT colonography. Of the six screenings, on average, participants reported 3.7 (±1.8) correctly.

Most participants correctly identified four of the six reasons prompting earlier screening (mean = 3.9 ± 1.5). The reasons that were least likely to be identified correctly were the new diagnosis of other cancer and new onset of abdominal symptoms. Almost all participants (88.0%) correctly identified colonoscopy as the next diagnostic step after a positive result.

### CRC screening perspectives/practices

Four out of five survey respondents felt that CRC screening is within the scope of Ob/Gyn practice (Table 3). Approximately 72% of respondents indicated that they used ACOG guidelines for screening, followed by about one in three who reported using the United States Preventive Services Task Force (USPSTF) guidelines. Of all CRC screening methods, more than four out of five participants reported using colonoscopy and about one in three use FOBT.

**Table 3.** Colorectal Cancer Screening Perspectives/Practices and Patient-Provider Interactions of Study Participants

Characteristic	*n*	%
Screening perspectives		
Colonoscopy contraindicated in pregnancy (no)	114	80.3
CRC screening within scope of practice (yes)	114	80.3
Screening practices		
Screening guidelines used^a^		
ACOG	102	71.8
USPSTF	45	31.7
ACG	21	14.8
ACP	5	3.5
Screening methods most used^a^		
Colonoscopy	123	86.6
Fecal occult blood test	45	31.7
Fecal immunohistochemical test	15	10.6
Flexible sigmoidoscopy	3	2.1
Double contrast barium enema	1	0.7
CT colonography	0	0.0
Patient-provider interactions		
Initiate CRC conversation		
Always	82	57.8
Most of the time	32	22.5
Some of the time/rarely/never	28	19.8
Patient barriers to CRC screening^a^		
Patient unwilling to undergo invasive procedure	107	75.4
Education/awareness	80	56.3
Financial	56	39.4
Lack of insurance coverage	45	31.7
Transportation	27	19.0
Most important barrier (MD opinion)		
Patient unwilling to undergo invasive procedure	82	60.7
Education/awareness	28	20.7
Financial	15	11.1
Lack of insurance coverage	6	4.4
Transportation	4	3.0
Approaches encouraging adherence to CRC screening (MD opinion)^a^		
Initiating discussion	109	76.7
Educational materials regarding		
Importance of CRC	73	51.4
Screening options	46	32.4
Mail reminders	36	25.4

^a^ Multiple responses possible so sums to >100%

ACG, American College of Gastroenterology; ACOG, American College of Obstetricians and Gynecologists; ACP, American College of Physicians; USPSTF, United States Preventive Services Task Force.

Respondents felt that the greatest patient barriers to CRC screening were patient's lack of willingness to undergo an invasive procedure (75.4%) and patient education and awareness (56.3%). Financial issues and lack of insurance coverage were felt to play less of a role (39.4% and 31.7%, respectively). Although half of participants felt that providing educational materials about the importance of CRC screening was helpful to address these barriers, three-quarters of respondents felt that initiating a discussion was the best approach to encourage adherence to CRC screening. Consistent with this, 80.2% report that they do initiate such discussions at least most of the time.

### Subset analyses: correct CRC knowledge

The number of items correct for CRC risk factors, CRC screening intervals, and reasons for earlier CRC screening was examined by respondent demographic and practice characteristics (Table 4). Comparisons for gender, age, and academic rank are shown in Table 4 (none of the other characteristics showed any significant differences in knowledge in these three areas). Females compared to males showed a pattern of identifying more correct items for all three variables, but the difference was significant only for screening intervals (4.0 ± 1.5 vs. 3.2 ± 2.0, *p* = 0.010, respectively).

**Table 4.** Correct Colorectal Cancer Knowledge of Study Participants by Age, Gender, and Academic Rank

Characteristic	*n*	Mean ± SD	*p*
Risk factors (correct)		5.3 ± 1.8	
Gender	130		0.171
Male	42	5.0 ± 2.1	
Female	88	5.5 ± 1.6	
Age	131		0.889
20–50	62	5.3 ± 1.5	
>50	69	5.3 ± 2.0	
Academic rank	124		0.042
Attending/fellow	93	5.3 ± 1.6	
Resident	20	5.9 ± 1.4	
Other	11	4.4 ± 1.2	
Screening interval (correct)		3.7 ± 1.8	
Gender	130		0.010
Male	42	3.2 ± 2.0	
Female	88	4.0 ± 1.5	
Age	131		<0.001
20–50	62	4.5 ± 1.5	
>50	69	3.1 ± 1.7	
Academic rank	124		0.002
Attending/fellow	93	3.7 ± 1.6	
Resident	20	4.8 ± 1.8	
Other	11	2.6 ± 1.4	
Earlier CRC screening (correct)		3.9 ± 1.5	
Gender	130		0.473
Male	42	3.7 ± 1.7	
Female	88	3.9 ± 1.3	
Age	131		0.011
20–50	62	3.5 ± 1.1	
>50	69	4.2 ± 1.6	
Academic rank	124		0.028
Attending/fellow	93	4.0 ± 1.4	
Resident	20	3.0 ± 1.1	
Other	11	3.5 ± 1.4	

The results for age of respondent were mixed. There was no difference in identifying risk factors, but younger respondents had significantly identified more screening intervals correctly compared to older respondents (4.5 ± 1.5 vs. 3.1 ± 1.7, *p* < 0.001, respectively). In contrast, older respondents identified more reasons for earlier screening correctly than those younger (4.2 ± 1.6 vs. 3.5 ± 1.1, *p* = 0.011, respectively).

Academic rank did have a significant relationship with knowledge for all three variables. Residents had a significantly higher mean number of items correct for risk factors and screening intervals followed by attendings/fellows and then the other group (risk factors: 5.9 ± 1.4 vs. 5.3 ± 1.6 vs. 4.4 ± 1.2, *p* = 0.042, respectively; screening interval: 4.8 ± 1.8 vs. 3.7 ± 1.6 vs. 2.6 ± 1.4, *p* = 0.002, respectively). Like age, the pattern reverses for reasons for earlier screening, where attendings/fellows identify the most correct reasons, followed by the other group and then residents (4.0 ± 1.4 vs. 3.5 ± 1.4 vs. 3.0 ± 1.1, *p* = 0.028, respectively).

### Subset analyses: CRC screening perspectives/practices

Screening perspectives and practices were assessed by the demographic and practice characteristics and in most cases, there were no significant differences (Table 5). There were some differences for age and academic rank and region and current practice (displayed in Table 5). Younger respondents were significantly more likely to use USPSTF screening guidelines than their older counterparts (48.4% vs. 18.8%, *p* < 0.001, respectively). They were also significantly less likely to use fecal occult blood testing (FOBT) (16.1% vs. 47.8%, *p* < 0.001, respectively). This pattern was also reflected in academic rank for use of the USPSTF guidelines, where residents were most likely to use these guidelines (65.0%), followed by the other group (45.5%), followed by attendings/fellows (23.7%) (*p* = 0.001).

**Table 5.** Screening Perspectives/Practices and Patient-Provider Interactions for Study Participants by Age, Academic Rank, US Region, and Current Practice

Characteristic	Age			Academic rank				Region				Current practice			
	% 20–50 years	% >50 years	*p*	% Attending	% Resident	% Other	*p*	% North East	% South	% Mid-West/West	*p*	% OB	% GYN	% OB/GYN	*p*
*n*	62	69		93	20	11		71	28	21		16	24	80	
Screening perspectives															
Colonoscopy contraindicated in pregnancy (no)	79.0	81.2	0.761	80.6	85.0	63.6	0.339	87.3	67.9	71.4	0.052	87.5	70.8	80.0	0.424
CRC screening within scope of practice (yes)	80.6	78.3	0.736	87.7	90.0	63.6	0.194	78.9	85.7	85.7	0.636	87.5	70.8	85.0	0.236
Screening practices															
Screening guidelines used^a^															
ACOG	79.0	65.2	0.079	76.3	75.0	72.7	0.961	74.6	78.6	81.0	0.805	75.0	75.0	78.8	0.898
USPSTF	48.4	18.8	<0.001	23.7	65.0	45.5	0.001	38.0	21.4	28.6	0.259	31.3	20.8	35.0	0.424
ACG	11.3	18.8	0.230	12.9	20.0	9.1	0.632	15.5	7.1	9.5	0.476	0.0	20.8	13.8	0.162
ACP	1.6	4.3	0.364	2.2	5.0	0.0	0.649	4.2	0.0	4.8	0.529	6.3	4.2	1.3	0.425
Screening methods used most^a^															
Colonoscopy	90.3	84.1	0.287	89.2	95.0	72.7	0.163	93.0	67.9	95.2	0.002	75.0	91.7	88.8	0.249
Fecal occult blood test	16.1	47.8	<0.001	28.0	15.0	63.3	0.015	19.7	60.7	28.6	<0.001	31.3	37.5	27.5	0.640
Fecal immunohistochemical test	6.5	14.5	0.137	12.9	5.0	18.2	0.500	8.5	25.0	4.8	0.038	0.0	12.5	15.0	0.254
Flexible sigmoidoscopy	0.0	4.3	0.097	2.2	0.0	0.0	0.713	0.0	3.6	9.5	0.045	0.0	0.0	3.8	0.463
Double contrast barium enema	1.6	0.0	0.290	1.1	0.0	0.0	0.845	1.4	0.0	0.0	0.706	6.3	0.0	0.0	0.038
Patient-provider interactions															
Initiate CRC conversation			0.499				0.169				0.063				<0.001
Always/most of the time	17.7	13.4		16.7	10.0	36.4		21.4	22.2	0.0		57.1	17.4	11.3	
Some of the time/rarely/never	82.3	86.6		83.3	90.0	63.6		78.6	77.8	100.0		42.9	82.6	88.7	
Patient barriers to CRC screening^a^															
Patient unwilling to undergo invasive procedure	83.9	72.5	0.116	75.3	90.0	81.8	0.335	84.5	67.9	76.2	0.173	75.0	66.7	82.5	0.241
Education/awareness	64.5	52.2	0.153	50.5	85.0	63.6	0.017	64.8	42.9	42.9	0.059	50.0	45.8	61.3	0.346
Financial	35.5	42.0	0.443	26.9	65.0	63.6	0.001	35.2	53.6	14.3	0.018	31.3	33.3	38.8	0.792
Lack of insurance coverage	24.2	36.2	0.135	28.0	25.0	45.5	0.438	19.7	53.6	38.1	0.003	31.3	33.3	30.0	0.952
Transportation	19.4	18.8	0.940	16.1	25.0	36.4	0.216	18.3	2.0	14.3	0.615	31.3	25.0	16.3	0.310
Most important barrier (MD opinion)			0.228				0.932				0.014				492
Patient unwilling to undergo invasive procedure	60.7	62.7		65.2	65.0	54.5		62.9	53.6	75.0		62.5	59.1	63.7	
Education/awareness	26.2	16.4		20.2	20.0	36.4		27.1	14.3	5.0		31.3	9.1	21.3	
Financial	4.9	13.4		9.0	5.0	9.1		4.3	25.0	10.0		6.3	18.2	10.0	
Lack of insurance coverage	3.3	6.0		3.4	5.0	0.0		2.9	7.1	0.0		0.0	9.1	2.5	
Transportation	4.9	1.5		2.2	5.0	0.0		2.9	0.0	10.0		0.0	4.5	2.5	
Approaches encouraging adherence to CRC screening (MD opinion)^a^															
Initiating discussion	74.2	81.2	0.338	78.5	65.0	81.8	0.395	73.2	82.1	90.5	0.207	62.5	79.2	76.3	0.444
Educational materials regarding															
Importance of CRC	54.8	50.7	0.638	51.6	70.0	36.4	0.164	56.3	42.9	52.4	0.481	50.0	45.8	57.5	0.565
Screening options	38.7	27.5	0.174	32.3	45.0	27.3	0.490	40.8	21.4	23.8	0.108	37.5	25.0	36.3	0.568
Mail reminders	29.0	21.7	0.337	23.7	40.0	9.1	0.137	22.5	28.6	28.6	0.755	25.0	16.7	27.5	0.561

^a^ Multiple responses possible, so sums to >100%

Ob/Gyn, obstetrician-gynecologist.

The use of FOBT showed a different pattern by academic rank, where a larger proportion of others used it (63.3%), followed by attendings/fellows (28.0%), followed by residents (15.0%) (*p* = 0.015). A significantly larger proportion of residents identified education/awareness as an important patient barrier to CRC screening compared to others or attendings/fellows (85.0% vs. 63.6% vs. 50.5%, *p* = 0.017, respectively). A similar significant pattern was seen with the identification of financial considerations as a barrier.

Regional differences emerged in the screening methods most used and the identification of patient barriers. A significantly smaller proportion of participants from the South used colonoscopy compared to those from the rest of the United States (67.9% vs. 93.0% [NE] vs. 95.2% [MW-W], *p* = 0.002). In contrast, Southern respondents were more likely to use FOBT (60.7% vs. 19.7% [NE] vs. 28.6% [MW-W], *p* < 0.001) and fecal immunohistochemical test (25.0% vs. 8.5% [NE] vs. 4.8% [MW-W], *p* = 0.038). Flexible sigmoidoscopy, on the contrary, was more likely to be reported by Midwest/West respondents compared to those from the South and Northeast [(9.5% vs. 3.6% [S] vs. 0.0% [NE], *p* = 0.045)].

Furthermore, a significantly larger proportion of Southern participants identified financial issues and lack of insurance coverage as barriers to CRC screening compared to the other regions (financial: 53.6% vs. 35.2% [NE] vs. 14.3% [MW-W], *p* = 0.018); lack of insurance (53.6% vs. 38.1% [MW-W] vs. 19.7% [NE], *p* = 0.003).

Finally, there were a few practice differences. A significantly larger proportion of obstetricians reported using double contrast barium enema compared to gynecologists and Ob/Gyns, who did not use them at all (6.3% vs. 0.0% (gynecology only and Ob/Gyn), *p* = 0.038). Also, significantly smaller proportions of gynecologists and Ob/Gyns reported initiating CRC screening conversations at least most of the time compared to obstetricians (11.3% [Ob/Gyns] vs. 17.4% [gynecologists] vs. 57.1% [obstetricians], *p* < 0.001).

## Discussion

The findings from this study show that most respondents were knowledgeable in some areas regarding CRC screening but not all. Most correctly identified the recommended age to begin screening (age 50 at the time) and when earlier screening was warranted. However, on average, they only identified half of the 10 risk factors listed and only one-quarter correctly identified the age at which screening can stop. That Ob/Gyns may have some gaps in their knowledge regarding CRC screening is consistent with other studies of medical trainees (including Ob/Gyns).^15^ The importance of physician knowledge in this area is the implications for adherence to CRC screening guidelines in practice.^16^

Although most study respondents felt that the main patient barrier to CRC screening was unwillingness to do an invasive procedure and patient awareness, they felt that the best ways to address these barriers was for physicians to initiate a discussion with their patients and to provide educational materials emphasizing the importance of screening. Similarly, several studies have indicated an increase in CRC screening if physicians were personally involved in making screening recommendations^11,17^ and have suggested that educational materials be made available to physicians to provide to their patients.^9^ It is interesting to note that obstetrician respondents were more likely to report initiating CRC screening discussions relative to Ob/Gyns and those providing only gynecologic care.

When comparing patient perceived barriers to CRC screening compared to provider perceived barriers, O'Malley et al^18^ describe patients' lack of knowledge about CRC screening, poor health literacy, negative attitudes about prevention and cancer, inconvenience, and the lack of physician recommendation for the testing as patient barriers. In addition, the authors describe historical mistrust of the medical system, and perceived bias in care delivery as barriers among Black patients.^18^ Low-income populations face social determinants of health as barriers, including lack of insurance, high cost of care, lack of childcare, job demands, or inadequate transportation, to the physician's office or colonoscopy site.^18^

In contrast, health care providers noted barriers to performing and ordering CRC screening for their patients; a lack of knowledge that there is randomized controlled trial evidence about the effectiveness of screening with FOBT in reducing CRC mortality; provider attitudes; and discouragement caused by the anticipated lack of patient cooperation and time pressures for counseling.^18^ Among providers who care for low-income and uninsured patients in safety net settings, the above barriers are often magnified, including lack of specialists in gastroenterology, long waits for appointments, and inability for appropriate referrals to specialists.

This work is one of the few studies that has investigated the knowledge and practices of hospital attendings compared to residents, specifically focused on Ob/Gyns. Our study observed that there was a difference in knowledge of colorectal risk factors and screening guidelines between residents and attendings in obstetrics and gynecology that can contribute to less than effective screening of CRC. Ob/Gyn residents (*i.e.,* those most recently trained) were most knowledgeable about screening guidelines for CRC. Similarly, those Ob/Gyns trained more recently were found by Menees and colleagues to be more likely to screen for CRC.^13^ Residents were also less likely to use FOBT, which parallels the movement toward colonoscopy in practice guidelines.^17^ In contrast, physicians practicing in the south were more likely to recommend FOBT. This, along with some other regional practice pattern differences noted, is of unclear clinical significance.

Resident knowledge of CRC screening guidelines was noted to be better than in other studies in the literature, which have demonstrated that overall accuracy of CRC screening ranged from 11% to 23% regardless of specialty. However, in a study by Patell et al,^16^ differences between specialties were shown in surveillance knowledge. Overall, among trainees in the United States, CRC screening and surveillance knowledge were poor.^16^

Ob/Gyns are uniquely positioned to offer preventive care services for their patients since they provide care throughout the lifespan. During the well-woman visit, Ob/Gyns may counsel their patients about preventive services to maintain healthy lifestyles and minimize overall health risks. In a shared decision-making model of care, Ob/Gyns can facilitate healthy behaviors and counsel regarding preventive health practices, such as screening for breast, cervical, and CRC.^19^

In addition to health care disparities in the community where patients may not know their risks and when to be screened for CRC, health care professionals have differences in knowledge about colorectal screening guidelines, which occur at different levels of training. A higher level of knowledge should be expanded not only among health care providers, but also in the community. Defining who needs to be evaluated and the evaluation modality indicated are of extreme importance to close these knowledge gaps.

Strengths of this study include that it is a more recent evaluation of the knowledge of screening practices among Ob/Gyns, especially including a larger proportion of African American providers than has typically been included in the past. This demographic emphasis may also pose a limitation in that the composition of the respondents, both demographically and geographically, is not reflective of the national composition. Since this is a convenience sample and the participants were drawn from several national meetings/organizations, this may somewhat limit the generalizability of the findings.

There are also other limitations to this study. Age and level of training were not independent variables so that more information might have been gleaned if the survey indicated year since training as opposed to the ages of the participants. Another limitation is that since the creation of this survey, screening practices may have changed based on guidelines of gastroenterologists. Finally, this study only assessed provider perspectives regarding barriers to screening. As shown by prior studies,^18^ patient views of barriers to CRC screening may differ.

## Conclusions

In summary, Ob/Gyns are critical to cancer screening in their patients. The role of well-woman care for the specialty cannot be understated. Increasing rates of CRC among younger populations have influenced the practice of Ob/Gyns in the screening and prevention of CRC.

## Supplementary Material

Supplemental data

## Supplementary Material

Supplementary Appendix A1

## Abbreviations Used

ACG: American College of Gastroenterology
ACOG: American College of Obstetricians and Gynecologists
ACP: American College of Physicians
BRANY: Biomedical Research Alliance of New York
CRC: colorectal cancer
CT: computed tomography
FIT: fecal immunochemical test
FOBT: fecal occult blood test
H+H: Health + Hospitals
NE/S/MW-W: Northeast/South/Midwest-West
NMA: National Medical Association
NYC: New York City
Ob/Gyn: obstetrician-gynecologist
SD: standard deviation
USPSTF: United States Preventive Services Task Force
