# Supplementary material for: Colorectal Cancer Screening Knowledge and Practices Among Practicing Obstetrician-Gynecologists and Residents
Source: Womens Health Rep (New Rochelle). 2023 Jan 5;4(1):1–10. doi: 10.1089/whr.2022.0065 (PMC9883669; doi:10.1089/whr.2022.0065)
Supplement: Supplemental data [file Suppl_AppendixA1.docx]

**APPENDIX A. A Survey Analysis: Colorectal Cancer Screening Practices Among OB/GYNs**

(Please note: Correct answers for Qs1-6 shown in red.)

1. Which of the following conditions may create an increased risk for colorectal cancer? [check all that apply]

❏ Familial adenomatous polyposis

❏ Juvenile Polyposis

❏ Diabetes mellitus

❏ Peutz-Jeghers

❏ Celiac Disease

❏ HNPCC - Lynch Syndrome

❏ Inflammatory bowel disease (Crohn’s Disease, Ulcerative Colitis)

❏ Inflammatory bowel syndrome

❏ Vegetarian diet

❏ First degree relative with colorectal cancer before age 60

2. For the average patient without risk factors, at what age would you recommend that colorectal cancer screening begin?

❏ 40

❏ 45

❏ 50

❏ 55

❏ 60

3. For the same patient, at what age would you recommend that screening end?

❏ 65

❏ 70

❏ 75

❏ 80

❏ 85

4. At what interval should each of the following screening methods be conducted? [please check 1 column for each screening method]

Method Yearly Every 5

years

Every 10

years

Colonoscopy: every 10 years

Flexible sigmoidoscopy: every 5 years

Fecal occult blood test: yearly

Fecal immunohistochemical testing: yearly

CT colonography: every 5 years

Double contrast barium enema: every 5 years

5. Which of the following situations would lead you to recommend earlier colorectal cancer screening for your patient? [Check all that apply]

❏ New onset abdominal symptoms

❏ Positive fecal occult blood test

❏ Change in stool pattern/frequency/appearance

❏ New diagnosis of other cancer in patient

❏ New diagnosis of inflammatory bowel disease in patient

❏ Family member with new colorectal cancer diagnosis

❏ Other __________________________________________

6. If positive results are found on a non-invasive screening method, what would be your next diagnostic step?

❏ Colonoscopy

❏ Abdominal CT

❏ Flexible sigmoidoscopy

❏ Abdominal ultrasound

❏ I’m unsure

❏ Other __________________________________________

7. In your opinion, would you say that colonoscopy is contraindicated in pregnancy?

Yes _______ No _______

Do you view colorectal cancer screening as within the scope of OB/GYN practice?

Yes _______ No _______

8. Which screening guidelines do you utilize within your practice?

❏ American Congress of Obstetricians and Gynecologists (ACOG)

❏ American College of Gastroenterology (ACG)

❏ American College of Physicians (ACP)

❏ US Preventative Services Task Force (USPSTF)

❏ Other _________________________________

9. Which screening methods are most commonly used/referred to within your practice?

❏ Colonoscopy

❏ Flexible sigmoidoscopy

❏ Fecal occult blood test

❏ Fecal immunohistochemical testing

❏ CT colonography

❏ Double contrast barium enema

❏ Other __________________________________

10. How often do you initiate a conversation about colorectal cancer screening with patients of appropriate age or with risk factors?

❏ Always

❏ Most of the time

❏ Some of the time

❏ Rarely

❏ Never

11. What are some common reasons that are barriers to your patients receiving colorectal cancer screening? [check all that apply]

❏ Financial barriers

❏ Lack of coverage by insurance

❏ Transportation to appointments

❏ Educational or awareness barriers

❏ Patient unwillingness to undergo invasive procedure

❏ Other: _____________________________________

12. What do you view as the most common barrier? [check one]

❏ Financial barriers

❏ Lack of coverage by insurance

❏ Transportation to appointments

❏ Educational or awareness barriers

❏ Patient unwillingness to undergo invasive procedure

❏ Other: _____________________________________

13. What approaches do you believe encourage adherence with colorectal cancer screening among your patients? [check all that apply]

❏ Mailed reminders to patients about their next screening due date

❏ Educational materials on the importance of colorectal cancer screening

❏ Educational materials detailing the various screening options

❏ Initiating an open discussion about colorectal cancer screening with every patient

❏ Other: __________________________________________________

14. Demographic data

Age: _______ Gender: M _____ F _____ Racial ethnicity: ____________________________

15. Current academic status:

❏ PGY1

❏ PGY2

❏ PGY3

❏ PGY4

❏ Fellow

❏ Attending

❏ Other _____ please specify: ______________________

16. Current workplace: _____________________________

City: ___________________ State: ________________

17. Current practice:

❏ Primarily gynecology

❏ Primarily obstetrics

❏ Both gynecology and obstetrics
